# High-Grade Urothelial Carcinoma with Clear-Cell (Glycogen-Rich) Features and Divergent Trophoblastic Differentiation: A Histopathological Case Report

**DOI:** 10.3390/reports9010003

**Published:** 2025-12-22

**Authors:** George Stoyanov, Dobri Marchev, Pavel Pavlov, Peter Ghenev, Hristo Popov

**Affiliations:** 1 Department of Pathology, Multiprofile Hospital for Active Treatment, 9700 Shumen, Bulgaria; 2 Department of Urology, Multiprofile Hospital for Active Treatment, 9700 Shumen, Bulgaria; 3 Department of Pathology, Complex Oncology Center, 9700 Shumen, Bulgaria; 4 Department of General and Clinical Pathology, Forensic Medicine and Deontology, Faculty of Medicine, Medical University—Varna, 9002 Varna, Bulgaria

**Keywords:** urothelial carcinoma, high-grade urothelial carcinoma, special subtype, divergent differentiation, clear cell (glycogen-rich) type, trophoblastic differentiation, differential diagnosis

## Abstract

**Background and Clinical Significance**: Urothelial carcinoma is one of the most commonly diagnosed malignant diseases. However, it has a much more favorable prognosis than other significantly less common malignancies. This statement, however, is true only for conventional urothelial carcinomas, not for those with divergent differentiation or a special type of urothelial carcinoma. **Case Presentation**: Herein, we present a case report of an 80-year-old female patient with multiple predominantly cardiovascular comorbidities and vascular dementia, who presented to our institution with genital bleeding. Clinical and diagnostic tests were difficult due to patient noncooperation; however, abdominal computer tomography and cystoscopy showed an advanced tumor originating from the ventral bladder wall. Histology of the tumor showed an invasive urothelial malignancy with foci of clear-cell (glycogen-rich) variant and dispersed, pleomorphic cells, which were immunohistochemically positive for beta-human chorionic gonadotropin. Hence, the diagnosis of high-grade urothelial carcinoma with clear-cell (glycogen-rich) morphology and divergent trophoblastic differentiation was established. Patient outcome was poor. **Conclusions**: While conventionally having a somewhat favorable prognosis, special subtypes and divergent differentiation in urothelial carcinomas, which warrant a high-grade diagnosis are not only rare but also highly aggressive conditions. Further challenges arise in their differential diagnosis with other advanced malignancies, which can develop in adjacent organs in both genders

## 1. Introduction and Clinical Significance

Nestled between uterine cervix and non-Hodgkin lymphoma malignancies, urothelial carcinoma is the ninth most commonly diagnosed malignancy overall as per the latest GLOBOCAN data [1]. The incidence in males is higher than in females, with a relatively low number of malignancy-related deaths in both genders. Urothelial carcinomas rank 13th overall, falling significantly behind rarer malignancies, such as pancreatic, esophageal, and central nervous system malignancies [1].

The lower mortality-to-incidence ratio can be explained by both the relatively high incidence of clinical symptoms in the early stages of development of urothelial carcinoma, predominantly gross hematuria and the easy and readily available access to urinary bladder endoscopy and biopsy, as well as urinary cytology testing without tissue biopsy [2,3]. Coupled together with the relatively high incidence of in situ papillary non-invasive (pTa) and papillary superficially invasive (pT1) urothelial carcinoma, compared to more advanced stages (pT2-4b) of urinary bladder urothelial carcinoma, the GLOBOCAN data reflects a generalization of this quite mixed and diverse group of malignancies [4].

While predominantly developing in the urinary bladder, urothelial carcinoma can also rarely develop in other parts of the urinary system, such as the renal pelvis, ureters, and urethra, where mortality is also higher [5,6]. Furthermore, other than tumor location and tumor stage, tumor grade is also an important survival factor, with the current classifications recognizing two levels of differentiation grades based off predominantly nuclear pleomorphism features—low-grade (less aggressive and prone to recurrence and progression) and high-grade (more aggressive and prone to recurrence and progression, typically present with higher stage as well) [7,8,9].

While histology of urothelial carcinoma is dominated by conventional urothelial morphology, wherein the above-mentioned characteristics of nuclear pleomorphism are used as grading criteria, there are several subtypes and divergent differentiations observed only in a minority of cases, wherein the designated grade is always high-grade, as these rare cases are associated with a more aggressive clinical behavior [10].

Herein, we present a case report of one such exotic variant of urothelial carcinoma of the urinary bladder, characterized by a unique morphology and aggressive behavior—urothelial carcinoma with trophoblastic differentiation.

## 2. Case Presentation

A polymorbid 80-year-old female patient presented to our institution with a history of several days of profuse genital bleeding and several days of mild abdominal pain. The patient, as already mentioned, had multiple comorbidities, predominantly cardiovascular, with a history of significant vascular dementia, as reported by her relatives. The patient’s previous gynecologic history included five normal pregnancies and deliveries. Physical and genital exams were difficult to perform as the patient was uncooperative. Both on palpation and ultrasound, the uterus was relatively enlarged (size referring to the first lunar month) and had a heterogeneous appearance on ultrasound. Vaginal exam, which was again difficult due to the patient being uncooperative, revealed a bloody discharge from the cervical canal. Bloodwork revealed significant anemia, with a hemoglobin level of 89 g/L, while other values were within the reference range.

As both the physical and ultrasound exams were limited, abdominopelvic computer tomography was performed under mild sedation. Computer tomography revealed a bladder formation with a density of 36 HU, measuring 43 × 28 mm (sagittal) and 43 × 38 mm (axial), originating from the ventral wall of the bladder. The formation showed post-contrast enhancement to 73 HU, primarily on the periphery. The lumen itself was filled with hyperdense hemorrhagic structures, equivalent to 55–60 Hounsfield units (HU), which did not change their density post-contrast or with gas, resulting in the formation of hydraeric levels.

As the genital bleeding was determined to be secondary to the bladder tumor formation, the patient was transferred to the urology department for bladder endoscopy with biopsy.

Bladder endoscopy under general anesthesia revealed an exophytic and invasive bleeding tumor on the anterior bladder wall. Several biopsies were performed from different areas; however, ablation was not performed due to excessive bleeding from the biopsy sites. Specimens sent for histopathology consisted of three grayish-white, firm fragments, with the largest measuring 18 × 5 mm.

**Figure 1:**
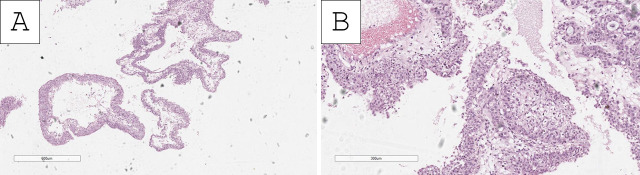
Histopathology of the tumor. (**A**) Foci of conventional-appearing urothelial papillary carcinoma, H&E stain, original magnification 40×; (**B**) foci of conventional-appearing papillary urothelial carcinoma admixed with focal clear-cell nests, H&E stain, original magnification 100×.

**Figure 2:**
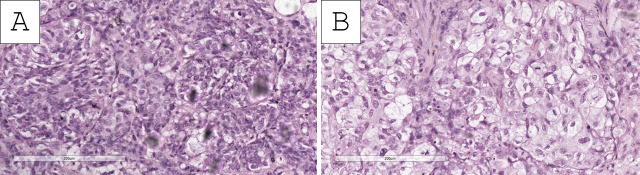
Histopathology of the tumor. (**A** Foci of conventional invasive urothelial carcinoma with focal clear cells, H&E stain, original magnification 200×; (**B**) predominantly clear-cell nests, H&E stain, original magnification 200×.

**Figure 3:**
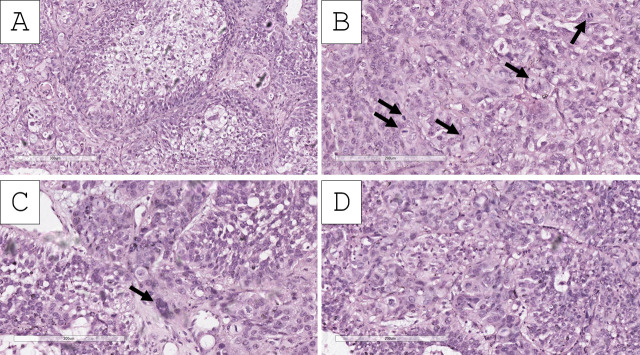
Histopathology of the tumor. (**A**) Clear-cell nests, H&E stain, original magnification 100×; (**B**) clear cells admixed with epithelioid type cells, pronounced mitotic activity (arrows), H&E stain, original magnification 200×; (**C**) epithelioid cell cords, some of which multinucleated (arrow), H&E stain, original magnification 200×; (**D**) epithelioid cell nests, H&E stain, original magnification 200×.

Histopathology of the specimens revealed fragments of urinary bladder wall with fibrovascular structures, characterized by nested submucosal and muscular infiltration, represented by large cellular aggregates with pronounced anisocytosis and anisokaryosis. These aggregates had uneven, hyperchromatic nuclei with multiple ruby-red nucleoli (Figure 1, Figure 2 and Figure 3). The tumor nest exhibited focal, abundant clear-cell transformation, characterized by double eosinophilic contouring of the nuclear outlines and nuclearly dominant cells with hyperchromatic nuclei and uneven borders (Figure 1, Figure 2 and Figure 3). Mitotic figures were abundant, including some pleomorphic ones with hotspots, showing up to three mitotic figures per single high-power field (400× magnification) (Figure 1, Figure 2 and Figure 3). Tumor cell emboli were present in the blood vessels.

**Figure 4:**
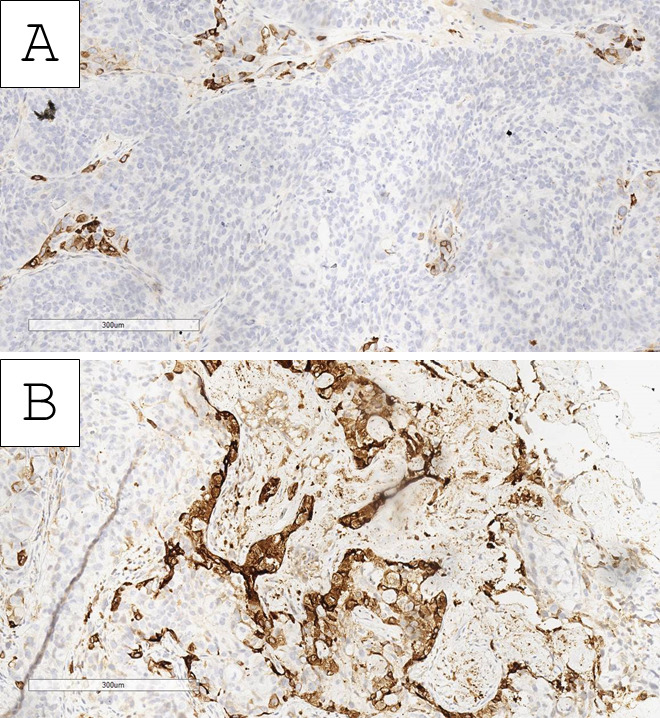
Immunohistochemistry with beta human chorionic gonadotropin. (**A**) Clear-cell nests negative and few intersecting zones with positive reaction, original magnification 200×; (**B**) predominantly positive nests, original magnification 200×.

Based on the morphological findings, the initial impression of the tumor was of a high-grade urothelial carcinoma with clear-cell (glycogen-rich) morphology and probable divergent differentiation (trophoblastic) or secondary specific histologic type (giant-cell urothelial carcinoma). Immunohistochemistry for beta human chorionic gonadotropin (hCG) showed an intense cytoplasmic reaction in the large, nuclearly dominant cells (Figure 4). Hence, the tumor was interpreted as a high-grade urothelial carcinoma with clear-cell (glycogen-rich) morphology and divergent trophoblastic differentiation. Staging was deemed to be at least pT2 due to invasion within the muscle wall with lymph and blood vessel tumor emboli.

The patient was referred to the oncology committee, which determined that the disease stage and concomitant conditions were contraindications for treatment, and the patient was referred for end-of-life palliative treatment and expired two months later due to disease progression and severe posthemorrhagic anemia.

## 3. Discussion

Urothelial carcinomas of special subtypes and divergent differentiation consistently warrant a high-grade designation, based on both their specific morphology and clinical behavior [10]. Specific subtypes are most often micropapillary and variants of nested urothelial carcinoma, with clear-cell (glycogen-rich) being a rare subtype [10]. In the presented case, the main morphological features are dominated by clear-cell (glycogen-rich) morphology. In this subtype, the cells accumulate cytoplasmic glycogen, which, due to the specifics of histological processing, is extracted in the final slide [10,11]. This special subtype is exceedingly rare, predominantly presented in case reports and small case series, and according to published data in the medical literature, it exhibits significantly more aggressive clinical behavior than conventional urothelial carcinoma [10,11,12,13]. This subtype also requires extensive differential diagnosis with one other subtype of urothelial malignancy—clear-cell adenocarcinoma of the urinary tract, which is a special type of Müllerian malignancy (PAX 8-positive, negative for GATA3, p63, ER, PR, and WT1), as well as clear-cell–renal-cell carcinoma (RCC-, PAX8- and CD10-positive) [12,13,14,15,16,17]. In females, this differential diagnosis is expanded to clear-cell carcinoma of the female genital (napsin A, WT1 and PR positive) tract and in males with clear-cell renal-type prostatic acinar adenocarcinoma (AMACR- and NKX3.1-positive) [11,13,14].

The presence of giant cells, especially nuclear-dominated ones, also warrants an extensive differential diagnosis, again with other exotic malignancies [10]. Urothelial carcinomas have both a special subtype with giant cells—giant-cell urothelial carcinoma and a divergent differentiation type—with trophoblastic differentiation, as seen in our case [10].

Giant-cell urothelial carcinoma is an aggressive subtype, also referred to as pleomorphic giant-cell carcinoma of the urinary bladder, represents an extreme spectrum of conventional high-grade urothelial carcinoma, represented by bizarre pleomorphic giant tumor cells in the background of at least minimal foci of conventional urothelial carcinoma, with the pleomorphic at least minimally retaining their urothelial immunohistochemical profile [18,19,20].

Conversely, in urothelial carcinoma with divergent trophoblastic differentiation, as seen in our case, the giant monstrous cells have a trophoblastic immunohistochemical phenotype and, in morphology, can be both cyto- and syncytotrophoblastic in nature to the point of the malignancy being hard to distinguish from choriocarcinoma [21,22,23]. This divergent type of urothelial carcinoma is also extremely rare and only reported in individual case reports and small case series and is often reported to coexist with special urothelial carcinoma subtypes and other forms of divergent differentiation [21,22,23].

Expression of trophoblastic markers in such cases is often not limited to cells of trophoblastic morphology, with hCG, hydroxyl-δ-5-steroid dehydrogenase and sal-like protein 4 being somewhat reliable markers for this divergent differentiation [21]. Of note is the presence of positivity of both hCG- and sal-like protein 4 in the conventional urothelial component of these malignancies, indicating the potential for divergent differentiation in urothelial malignancies [21]. This would also explain the clinical phenomenon of elevated serum hCG in patients with urothelial carcinoma, especially in advanced stages, and its potential to be used as a clinical marker for disease follow-up and progression [24,25,26].

Differential diagnosis, especially in cases with extensive divergent trophoblastic differentiation, is that of choriocarcinoma and other rare pleomorphic malignancies of the urogenital tract, such as giant-cell pleomorphic acinar adenocarcinoma of the prostate in males [21,27].

As seen in the outcome of our case, divergent trophoblastic differentiation in urothelial carcinomas is associated with an aggressive clinical course and poor patient outcome [21].

## 4. Conclusions

Divergent trophoblastic differentiation is a rare occurrence in urothelial carcinoma and warrants a diagnosis of high-grade malignancy. Although exceedingly rare and often posing a diagnostic challenge, this differentiation should always be kept in mind when diagnosing highly pleomorphic urothelial carcinomas, especially in the presence of other special types of divergent differentiations, as it is associated with a more aggressive clinical course when compared to conventional urothelial carcinoma, as underlined by the poor outcome of the presented case.

## Data Availability

The original contributions presented in this study are included in the article. Further inquiries can be directed to the corresponding author.
